# Intranasale Lidocainvernebelung als neue und nichtinvasive Therapieoption des Postpunktionskopfschmerzes: Zwei Fallberichte aus der geburtshilflichen Anästhesiologie

**DOI:** 10.1007/s00101-020-00900-9

**Published:** 2020-12-10

**Authors:** Benedikt Hermann Siegler, Marco Gruß, Beatrice Oehler, Jens Keßler, Herbert Fluhr, Claudia Weis, Frank Schulz, Markus Alexander Weigand

**Affiliations:** 1 Klinik für Anästhesiologie, Universitätsklinikum Heidelberg, Im Neuenheimer Feld 420, 69120 Heidelberg, Deutschland; 2 Klinik für Anästhesiologie, operative Intensivmedizin und Schmerztherapie, Klinikum Hanau GmbH, Leimenstraße 20, 63450 Hanau, Deutschland; 3 Klinik für Frauenheilkunde und Geburtshilfe, Universitätsklinikum Heidelberg, Im Neuenheimer Feld 440, 69120 Heidelberg, Deutschland

**Keywords:** Geburtshilfe, Regionalanästhesie, Postpartale Komplikationen, Lokalanästhetika, „Mucosal atomization device“, Obstetrics, Regional anesthesia, Postpartal complications, Local anesthetics, Mucosal atomization device

## Abstract

**Hintergrund:**

Der Postpunktionskopfschmerz („postdural puncture headache“ [PDPH]) stellt eine ernsthafte anästhesiologische Komplikation geburtshilflich behandelter Patientinnen dar. Führen konservativ-medikamentöse Therapieversuche nicht zu einer Symptomlinderung, empfehlen aktuelle Leitlinien die frühzeitige Durchführung eines epiduralen Blut-Patch. Als potenzielle Alternative wird die transnasale Blockade des Ganglion sphenopalatinum mittels Lokalanästhetika diskutiert.

**Methode:**

In dieser Falldarstellung wird erstmals von einer Modifikation dieser Technik unter Anwendung eines Medikamentenzerstäubers („mucosal atomization device“ [MAD]) zur Therapie eines PDPH bei zwei geburtshilflichen Patientinnen berichtet. Über dieses Verfahren existieren bislang keine Erfahrungen aus der geburtshilflichen Anästhesiologie.

**Ergebnisse:**

Die erste Patientin (25-jährige Zweitgravida, BMI 54,7 kg/m^2^) zeigte am ersten Tag nach Sectio caesarea in Spinalanästhesie einen ausgeprägten PDPH mit starker Übelkeit und Erbrechen. Bei der zweiten Patientin (32-jährige Drittgravida, BMI 27,3 kg/m^2^) kam es 4 Tage nach Spontanpartus unter Periduralanästhesie zu einer PDPH-bedingten Wiederaufnahme. Während konservative Maßnahmen sowie Therapieversuche mit Nichtopioidanalgetika und Koffein keinen hinreichenden Behandlungserfolg erzielten, führte die intranasale Lidocainapplikation mittels MAD zu einer unmittelbaren und persistierenden Linderung der Beschwerden. Von beiden Patientinnen wurde die Lidocaingabe sehr gut vertragen; sie konnten am Folgetag aus dem Krankenhaus entlassen werden.

**Schlussfolgerung:**

Die vorgestellte nichtinvasive und einfach durchzuführende Maßnahme stellt eine wertvolle Ergänzung bisheriger Therapieoptionen und eine potenzielle Alternative zum epiduralen Blutpatch bei geburtshilflichen Patientinnen mit PDPH dar.

## Abstract (en)

**Background:**

Postdural puncture headache (PDPH) occurs in up to 11% of patients after spinal anesthesia and in more than 80% after dural perforation upon epidural anesthesia. It represents a severe anesthesiological complication in obstetric patients. If conservative medication measures do not result in a timely relief of symptoms, the current guidelines recommend the early implementation of an epidural blood patch; however, although performing an epidural blood patch is effective to treat PDPH, potential side effects include neurological complications, spinal hematoma and infections. Assumed to reduce cerebral vasodilatation as a potential pathophysiological driver of PDPH, the transnasal block of the sphenopalatine ganglion with local anesthetics is discussed as an alternative approach.

**Methods:**

In this case study a modification of this technique is reported using a mucosal atomization device (MAD) for off-label nasal administration of lidocaine in two obstetric patients suffering from PDPH. Up to now there is no experience with this modified technique in obstetric anesthesiology.

**Results:**

The first patient (25-year-old secundigravida, body mass index [BMI] 54.7 kg/m^2^) displayed a pronounced PDPH with nausea and vomiting during the first day after a cesarean section under spinal anesthesia (3 attempts). The second patient (32-year-old tertiagravida, BMI 27.3 kg/m^2^) was readmitted to hospital due to PDPH 4 days after a natural birth under epidural anesthesia. Whereas conservative measures and therapeutic attempts with nonopioid analgesics and caffeine did not result in a sufficient treatment success, intranasal lidocaine administration via a MAD led to an immediate and persisting symptom relief. Both patients could be discharged from hospital after 24 h of surveillance and did not report any relevant side effects of the lidocaine administration.

**Conclusion:**

The described noninvasive and simple procedure represents a valuable addition to previously known treatment options for PDPH and a potential alternative to an epidural blood patch in obstetric patients with PDPH. Prospective studies are needed to validate the findings.

## Hintergrund

Bei ca. 22 % aller schwangeren Patientinnen in Deutschland werden zur Milderung des Geburtsschmerzes rückenmarknahe Anästhesieverfahren eingesetzt [1]. Zu den typischen Komplikationen dieser Verfahren gehört der Postpunktionskopfschmerz („postdural puncture headache“ [PDPH]) nach einer Spinalanästhesie (SpA) oder infolge einer akzidentiellen Punktion der Dura mater im Rahmen einer Periduralanästhesie (PDA). Mit einer Häufigkeit zwischen 1,5 und 11,2 % nach SpA [2] resp. >80 % nach ungewollter Duraverletzung während einer PDA [3] stellt der PDPH eine nichtunerhebliche Belastung der betroffenen Patienten dar. Hierbei weisen Frauen ein gegenüber Männern deutlich erhöhtes PDPH-Risiko auf [4].

In der überwiegenden Mehrheit der Fälle tritt ein PDPH innerhalb von 3 Tagen nach stattgehabter Punktion auf [5], wobei potenziell auch ein deutlich verspäteter Beginn der Symptomatik möglich ist [6]. Entgegen der ursprünglichen Annahme, dass es sich bei einem PDPH um ein selbstlimitierendes Phänomen handelt, kommt es nicht selten zu einer Chronifizierung des Schmerzes [7]. Bei mehr als einem Drittel aller Betroffenen führen die Beschwerden zu einer deutlichen Einschränkung der Leistungsfähigkeit [8]. Im Bereich der Geburtshilfe nimmt dies nicht nur direkten Einfluss auf die mütterliche Versorgung des Neugeborenen, sondern kann bei Folgeschwangerschaften zu einer ablehnenden Haltung der Frauen gegenüber rückenmarknahen Anästhesieverfahren beitragen [9].

Wenngleich die zugrunde liegende Pathophysiologie noch nicht abschließend geklärt ist, werden dem Verlust von Liquor und der kompensatorischen intrakraniellen Vasodilatation eine zentrale Bedeutung beigemessen. Zudem kann es durch den Liquorverlust zu einem Zug an Nerven, Gefäßen und einer Reizung der Hirnhaut selbst kommen, weshalb die ausgelösten Symptome typischerweise durch eine aufrechte Körperposition verstärkt werden [5].

Die klinischen Charakteristika und diagnostischen Kriterien des PDPH sind in Tab. 1 zusammengefasst. Bei Zweifel an der klinischen Diagnose/atypischer Klinik sowie bei persistierenden Beschwerden sollte laut der aktuellen S1-Leitlinie *Die geburtshilfliche Analgesie und Anästhesie* der Deutschen Gesellschaft für Anästhesiologie und Intensivmedizin in Zusammenarbeit mit der Deutschen Gesellschaft für Gynäkologie und Geburtshilfe eine differenzialdiagnostische Abklärung (frühzeitig bildgebende Verfahren sowie die Durchführung eines neurologischen Konsils; Tab. 1) erfolgen [1].

**Table 1:** 

**Klinische Charakteristika**	*Auftreten bzw. Verschlechterung innerhalb von 15* *min nach dem Aufrichten sowie mindestens eines der folgenden Symptome:* Nackensteifigkeit Tinnitus Veränderung des Hörens Fotophobie Nausea
	*Vorausgegangene Liquorpunktion*
	*Auftreten innerhalb von 5 Tagen nach Liquorpunktion*
	*Remission spontan oder innerhalb von 48* *h nach effektiver Therapie*
**Bei anhaltender oder atypischer Klinik**	*Bildgebung* Computertomographie Magnetresonanztomographie
	*Neurologisches Konsil*
**Mögliche Differenzialdiagnosen**	*Perfusionsstörung* Intrazerebrale Blutung/subdurales Hämatom Hirnvenenthrombose Apoplex Hypophysenischämie
	*Intrakranieller Tumor*
	*Sonstiges* Virale, chemische oder bakterielle Meningitis Migräne Koffeinentzugskopfschmerz Drogenentzug Präeklampsie Spontanes Liquorunterdrucksyndrom Hypovolämie Pneumozephalus Laktationskopfschmerz

Zur Behandlung des PDPH kommen zunächst konservative Maßnahmen oder medikamentöse Therapieversuche beispielsweise mit Nichtopioidanalgetika oder Koffein zur Anwendung ([1]; Tab. 2). Führen diese nicht innerhalb kurzer Zeit zu einer Linderung der Beschwerden, wird die frühzeitige Beratung der Patientin hinsichtlich der Durchführung eines epiduralen Blut-Patch (EBP) empfohlen [1]. Dies ist auch insofern von Relevanz, als dass Frauen mit PDPH eine erhöhte Inzidenz einer postpartalen Sinusvenenthrombose oder eines Subduralhämatoms aufweisen [11]. Bezüglich dieser Komplikation ist eine prophylaktische Wirkung durch Sistieren des Liquorverlustes nach Anlage eines EBP potenziell denkbar.

**Table 2:** 

Substanz	Dosierung
Koffein	3- bis 4‑mal 200–300 mg/Tag p.o.
Theophyllin	3‑mal 280–350 mg/Tag p.o.
Gabapentin	Ein- bis 4‑mal 300 mg/Tag p.o.
Hydrocortison	Ein- bis 3‑mal 10 mg/Tag p.o.

Entgegen weit höherer Erfolgsraten nach Durchführung eines EBP in älteren Untersuchungen zeigen neuere Studien eine komplette und andauernde Beschwerdelinderung in ca. einem Drittel der untersuchten Fälle, während eine zumindest teilweise Besserung in 50–80 % erzielt wird [10]. Damit wird der EBP weiterhin als effektive Therapie des PDPH angesehen, allerdings geht dieses invasive Verfahren per se ebenfalls mit dem Risiko teilweise schwerwiegender Komplikationen wie der Ausbildung von subduralen Hämatomen, Nervenschädigungen, spinalen Infektionen oder einer weiteren Verschlimmerung der bestehenden Symptomatik einher [11, 12].

Als potenzielles Alternativverfahren zum EBP wird die transnasale Blockade des Ganglion sphenopalatinum diskutiert [13, 14], welche bereits bei verschiedenen Formen des Kopfschmerzes eingesetzt wird [15]. Hierbei wird den Patienten ein mit Lokalanästhetikum getränkter Watteträger an der Oberkante der Concha nasalis media entlang in Richtung Nasopharynxhinterwand eingeführt und für einige Minuten im Bereich des Ganglions auf Höhe der Fossa pterygopalatina platziert [16]. Die exakten Mechanismen, über die eine topische Blockade des Ganglion sphenopalatinum zu einer Beschwerdelinderung führt, sind bislang nicht bekannt. Eine mögliche Theorie beinhaltet jedoch die Blockade parasympathischer Ganglionanteile, welche einer intrakraniellen Vasodilatation als Treiber der PDPH-Symptomatik entgegenwirkt [17]. Potenzielle Nebenwirkungen umfassen temporäre nasopharyngeale Missempfindungen, Geschmacksstörungen, Irritationen der Schleimhaut sowie Nasenbluten.

In diesen Fallberichten wird erstmals die Anwendung einer Modifikation dieser Technik zur Therapie des PDPH bei geburtshilflichen Patientinnen beschrieben. Beide Patientinnen wurden zwischen Januar und Juni 2020 am Universitätsklinikum Heidelberg sowie am Klinikum Hanau behandelt. Nach Aufklärung über die Off-label-Nutzung wurde Lidocain 2 % mittels „mucosal atomization device“ (MAD) intranasal vernebelt. Über dieses Verfahren existieren bislang keine Erfahrungen aus der geburtshilflichen Anästhesiologie.

## Fallbeschreibung 1

### Anamnese

Bei der ersten Patientin handelte es sich um eine 25-jährige Zweitgravida (Tab. 3), welche zur geplanten Re-Sectio caesarea in SpA bei rascher Schwangerschaftsfolge in der 37 + 1 SSW stationär aufgenommen wurde. Die Durchführung der SpA wurde als erschwert dokumentiert; nach insgesamt 2 Punktionsversuchen auf 2 Höhen mit einer 25-G-Sprotte-Nadel gelang schließlich mit einer 12 cm langen 22-G-Sprotte-Nadel die Punktion des Liquorraums. Nach komplikationslosem Eingriff konnte die Patientin in den Kreißsaal und nach Abklingen der SpA auf die Normalstation verlegt werden.

**Table 3:** 

	Patientin 1	Patientin 2
*Alter (Jahre)*	25	32
*Körpergewicht (kg)*	140	70
*BMI (kg/m*^*2*^*)*	54,7	27,3
*Schwangerschaftswoche*	37 + 1	38 + 5
*Geburtshilfliche Anästhesie*	Spinalanästhesie	Periduralanästhesie
Nadeltyp	Sprotte	Tuohy
Nadelgröße	Initial 25 G, dann 22 G	18 G
*PDPH-Symptome*		
Lageabhängigkeit	Ja	Ja
Nackensteifigkeit	Nein	Ja
Tinnitus	Nein	Ja
Dysakusis	Nein	Ja
Photophobie	Nein	Nein
Nausea	Ja	Nein
*Therapie*		
Initial	4‑mal 600 mg Ibuprofen 4‑mal 1 g Paracetamol 3‑mal 200 mg Koffein	4‑mal 600 mg Ibuprofen 4‑mal 1 g Paracetamol 3‑mal 200 mg Koffein
Lidocain, intranasal	Einmal via getränktem Tupfer (ca. 50 mg/Seite) Einmal 50 mg/Seite (MAD)	100 mg/Seite (MAD)
Zeit Lidocaingabe via MAD bis Symptomlinderung (min)	5	2
NRS vor Lidocaingabe	9/10	7/10
NRS nach Lidocaingabe	0/10–1/10	0/10

*BMI* body mass index, *PDPH* postdural puncture headache, *MAD* mucosal atomization device, *NRS* numeric rating scale

### Befund und Diagnose

Am Morgen des ersten postoperativen Tages klagte die Patientin über lageabhängige stärkste Kopf- und Nackenschmerzen mit Ausbreitung über den Rücken bis ins Gesäß, begleitet von Schwindelgefühl sowie ausgeprägter Nausea und Emesis. Bereits geringe Oberkörperhochlagerung führte zu einer deutlichen Verstärkung der Symptomatik; ein aufrechter Gang war nicht möglich. Es bestanden keine neurologischen Defizite; die Einstichstellen zeigten sich unauffällig. In Zusammenschau der Befunde wurde die Diagnose eines PDPH nach einer erschwerten SpA gestellt.

### Therapie und Verlauf

Die symptomatische Therapie wurde um Ibuprofen, Koffein und Dimenhydrinat ergänzt (Tab. 3). Bei ausbleibender Beschwerdelinderung wurden mit der Patientin weitere Therapiemöglichkeiten, inklusive EBP, eruiert; die Patientin entschied sich nach Aufklärung über die Off-label-Gabe zunächst für die beidseitige nasale Einlage lidocaingetränkter Watteträger (ca. 50 mg/Seite). Auch hierunter besserten sich die Beschwerden nur unzureichend. Mit der Patientin wurde daraufhin die Möglichkeit einer weiteren Therapieeskalation mittels EBP besprochen. Bei weiterbestehenden Beschwerden wurde als alternativer Therapieversuch die Lidocainvernebelung mittels MAD angeboten, welcher die Patientin zustimmte. Vier Stunden nach Einlage der lidocaingetränkten Wattetupfer erfolgte die einmalige Vernebelung mittels MAD von jeweils 50 mg Lidocain/Seite. Hierunter zeigte sich die Patientin nach ca. 5 min nahezu symptomfrei; lediglich in aufrechter Position persistierten noch minimale Schmerzen (NRS 1/10). Diese bildeten sich im weiteren Verlauf ebenfalls vollständig zurück, sodass die Patientin am Folgetag schmerz- und beschwerdefrei nach Hause entlassen werden konnte.

## Fallbeschreibung 2

### Anamnese

Die zweite Patientin (32-jährige Drittgravida) wurde bei vorzeitigem Blasensprung aufgenommen. Unter als problemlos dokumentierter Periduralanästhesie (Anlage mit einer 18-G-Tuohy-Nadel, ein Punktionsversuch) kam es zunächst zu einer komplikationslosen Spontangeburt. Die vollständige Entfernung des Katheters erfolgte bei geplanter ambulanter Behandlung ca. 2 h post partum.

### Befund und Diagnose

Zum Zeitpunkt der Katheterentfernung bestand eine leichte Verspannung im Nackenbereich. Weitere 2 h später beklagte die Patientin eine ausgeprägte Nackensteifigkeit, Nackenschmerzen, Bewegungseinschränkungen beider Arme und Schultern sowie im Verlauf starke Kopfschmerzen, wobei eine Lageabhängigkeit der genannten Symptome bestand (Tab. 3). Mit Beginn der Beschwerden wurde neben der symptomatischen Therapie ein neurologisches Konsil initiiert. Hierbei wurde der Verdacht auf Vorliegen eines PDPH, differenzialdiagnostisch einer muskulären Verspannung oder einer meningealen Reizung nach PDK-Anlage gestellt.

### Therapie und Verlauf

Trotz leichter Symptomverbesserung unter angepasster Schmerzmedikation (Tab. 3) erfolgten die Aufklärung über eine potenzielle Therapieeskalation (inklusive EBP) sowie am Folgetag eine cMRT-Diagnostik, welche keine pathologischen Befunde ergab. Bei unauffälliger Bildgebung und zunächst weiterer Beschwerdebesserung wurde die Patientin am ersten postpartalen Tag auf eigenen Wunsch nach Hause entlassen.

Im weiteren Verlauf kamen zu den beschriebenen Symptomen ein Tinnitus sowie eine deutliche Hörminderung beidseits hinzu, sodass sich die Patientin am vierten postpartalen Tag erneut in der Klinik vorstellte, um das postpunktionelle Syndrom via Blut-Patch therapieren zu lassen. Hierüber wurde sie nun auch schriftlich aufgeklärt. Aufgrund der über mehrere Tage fortgeführten regelmäßigen Ibuprofeneinnahme wurde ein EBP jedoch nicht am selben Tag durchgeführt. Daher erfolgte bei hohem Leidensdruck nach ausführlicher Aufklärung über die Off-label-Gabe die fraktionierte nasale Applikation von jeweils 100 mg Lidocain/Seite via MAD. Die Option der Anlage eines Blut-Patch am Folgetag bei ausbleibender oder ungenügender Wirkung wurde durch Pausieren der Ibuprofeneinnahme sichergestellt. Nach intranasaler Lidocainvernebelung kam es innerhalb von ca. 2 min zu einem kompletten Sistieren der Kopf- und Nackenschmerzen sowie des Tinnitus; lediglich die Hörminderung blieb bestehen. Am Folgetag war die Patientin schmerzfrei; Tinnitus und Hörvermögen zeigten sich deutlich gebessert, mit lediglich noch vorhandener linksseitiger Einschränkung. Die Patientin konnte schließlich am ersten Tag nach Wiederaufnahme (fünfter postpartaler Tag) in deutlich gebessertem Allgemeinzustand nach Hause entlassen werden. Als einzige Nebenwirkung wurde ein vorübergehendes pharyngeales Taubheitsgefühl berichtet. Die Patientin wurde im weiteren Verlauf noch mehrfach kontaktiert; die noch geringfügig zum Zeitpunkt der Entlassung vorhandenen linksbetonten Einschränkungen des Hörvermögens sowie der Tinnitus verbesserten sich zunehmend und waren ca. 5 Wochen nach Punktion komplett verschwunden.

## Diskussion

In diesem Fallbericht wird erstmals der erfolgreiche Einsatz einer intranasalen Lidocainvernebelung mittels MAD zur Therapie eines PDPH nach geburtshilflicher rückenmarknaher Anästhesie geschildert. Bei beiden Patientinnen kam es nach erfolglosen Therapieversuchen mit Nichtopioidanalgetika sowie Koffein durch intranasale Lidocainverabreichung zu einer rasch eintretenden und anhaltenden Symptomlinderung.

Die hier beschriebene Methode weist unseres Erachtens einige Vorteile gegenüber bislang beschriebenen Verfahren zur Blockade des Ganglion sphenopalatinum auf. So kann das Einbringen von Watteträgern aufgrund der anatomischen Lokalisation insbesondere bei Patienten mit anatomischen Besonderheiten wie Polypen oder Septumdeviation erschwert sein, was den ausgebliebenen Therapieeffekt in unserem erstgenannten Fall erklären könnte. Interessanterweise benötigte unsere Patientin zur Einlage der Watteträger keine vorherige Lokalanästhesie, wohingegen in der Literatur teilweise ein Eintröpfeln oder -sprühen von Lokalanästhetika vor Durchführung der eigentlichen Prozedur beschrieben wird [18, 19]. Da das Ganglion sphenopalatinum von einer nur wenige Millimeter messenden Schleimhautschicht überdeckt wird [20], ist nicht auszuschließen, dass durch eine solche Vorabverabreichung von Lokalanästhetika bereits eine (Teil)Wirkung erzielt werden konnte.

Einen aus unserer Sicht wichtigen Aspekt im Vergleich zwischen konventionellen nasalen Blockadeformen und der hier beschriebenen Lokalanästhetikavernebelung stellt der Komfort für die Patientinnen während der Anwendung dar. Während das Einbringen von Watteträgern und deren Verbleib im Nasopharynx über den Zeitraum der Prozedur als sehr unangenehm empfunden werden können, sodass für die Durchführung in Einzelfällen eine Sedierung nötig ist [19], wird das Vernebeln des Lokalanästhetikums als vergleichbar zur Anwendung eines Nasensprays beschrieben. Als mögliche Nebenwirkung kann es durch Schlucken der Lokalanästhetikalösung jedoch zu temporären pharyngealen Missempfindungen kommen. In den hier berichteten Fällen wurde fraktioniert Lidocain 2 % vernebelt; zur Vermeidung der genannten Nebenwirkung wäre daher der zukünftige Einsatz geringerer Mengen höher konzentrierter Lösungen denkbar. So beschreiben Kanai et al. die erfolgreiche Anwendung 8 %igen Lidocainsprays zur Therapie einer Trigeminusneuralgie [21]. Gegenüber vorgefertigten Spraysystemen ermöglichen MAD-Systeme eine individuell angepasste Dosierung. Dies ist insofern relevant, da systemische Nebenwirkungen durch Resorption des intranasal applizierten Lidocains bei unsachgemäßer Handhabung und Überschreitung toxischer Grenzwerte prinzipiell möglich sind. Die von uns eingesetzte Dosis lag jeweils unterhalb der maximalen Einzeldosierung von 300 mg; bei beiden Patientinnen kam es zu keinen systemischen Nebenwirkungen.

Zusammenfassend handelt es sich bei der vorgestellten Maßnahme um eine aus unserer Sicht wertvolle Ergänzung bisheriger Therapieoptionen und eine potenzielle minimal-invasive Alternative zum epiduralen Blutpatch. Die Vernebelung von Lokalanästhetika via MAD ist eine bettseitig durchführbare, kostengünstige und mit einem deutlichen Zugewinn an Patientenkomfort verbundene Methode, deren Anwendung nicht nur bei PDPH in der Geburtshilfe sinnvoll sein kann. Es gilt zu beachten, dass es sich bei der intranasalen Verneblung von Lidocain um eine *Off-label*-Anwendung handelt, über die entsprechend aufgeklärt und dies auch dokumentiert werden muss. Prospektive Studien sind nötig, um die hier berichteten Erkenntnisse zu validieren und Aussagen zu optimaler Dosierung und Wirksamkeit auch anderer Lokalanästhetika im Rahmen der transnasalen Applikation bei PDPH treffen zu können [22].

## Fazit für die Praxis

- Es wird erstmals der Einsatz einer nasalen Lidocainvernebelung mittels Mucosal atomization device (MAD) zur Therapie eines Postpunktionskopfschmerzes (PDPH) nach geburtshilflicher rückenmarknaher Anästhesie beschrieben.
- Nach vorangegangen frustranen konventionell-medikamentösen Therapieversuchen führte die nasale Lidocaingabe zu einer unmittelbaren und persistierenden Besserung der Symptome; die Anlage eines epiduralen Blutpatch (EBP) konnte in beiden Fällen vermieden werden.
- Die geschilderte Methode ist einfach und bettseitig durchführbar, mit einem deutlichen Zugewinn an Patientenkomfort verbunden und sollte als neue potenzielle Therapieoption des PDPH und nichtinvasive Alternative zum EBP prospektiv validiert werden.
